# Near resolution of cutaneous lesions in long standing refractory dermatomyositis with deucravacitinib: A case report and literature review

**DOI:** 10.1016/j.jdcr.2026.02.031

**Published:** 2026-02-19

**Authors:** Christopher Alihosseini, Alyssa Forsyth, Hannah Kopelman, Brad P. Glick

**Affiliations:** a Dermatology, Larkin Community Hospital Palm Springs Campus, Hialeah, Florida; b Texas College of Osteopathic Medicine, Fort Worth, Texas; c KCU-GME Consortium/ADCS Orlando Program, Advanced Dermatology and Cosmetic Surgery, Maitland, Florida

**Keywords:** Dermatomyositis, Deucravacitinib, Refractory, Sotyktu

## Introduction

Dermatomyositis (DM) is an idiopathic autoimmune disorder characterized by chronic inflammation of the skin and skeletal muscle. The classic myopathic form presents as symmetrical weakness in the proximal muscles, usually affecting the shoulder and pelvic girdles, along with skin manifestations. Pathognomonic skin findings include Gottron papules and heliotrope rash, but other skin features such as shawl sign, V-sign, calcinosis, mechanic’s hands, and telangiectasias may also be seen.^1^

Current standard of care for DM with muscle involvement includes initial treatment with systemic corticosteroids, such as prednisone.^1^ Methotrexate (MTX), azathioprine, and tacrolimus may be administered concurrently or as second-line steroid-sparing agents.^1^ Mycophenolate mofetil (MMF) is an option, typically considered for patients with associated interstitial lung disease.^1^ Intravenous immunoglobulin (IVIG) is approved by the Food and Drug Administration (FDA) for moderate-to-severe or refractory disease in DM.^2^ Anifrolumab, a monoclonal antibody that inhibits the type-1 interferon receptor, has also showed rapid, multisystem efficacy in DM patients with both cutaneous and muscular function manifestations.^3^ There is also increasing evidence for the role of Janus kinase (JAK) inhibitors in the management of DM, especially in refractory cases, as demonstrated in the case reports by Lapa and Breslavets as well as McNamara and Tjahjono (Table I).^4^^,^^5^ Here, we present a case of classic myopathic DM in which cutaneous disease was successfully managed with deucravacitinib (Sotyktu, Bristol Myers Squibb, USA), a selective tyrosine kinase 2 (TYK2) inhibitor, as well as 2 other cases reported in the literature.

**Table I:** Demographics, clinical features, and outcomes of reported cases involving deucravacitinib therapy in patients with dermatomyositis

Study name	Patient age and sex	DM presentation	Treatment	Outcome
Lapa and Breslavets^4^	50-y-old woman	3 year history of clinically amyopathic DM with persistent facial edema, heliotrope rash, violaceous erythema, and Gottron sign	Failed: MTX, MMF, HCQ, topical and oral roflumilast Responded to deucravacitinib 6 mg daily	Complete near-resolution of cutaneous lesions within 10 wk; response remained stable at 6 mo; continuing treatment
McNamara and Tjahjono^5^	60-y-old man	6 month history of amyopathic DM with heliotrope rash and violaceous poikilodermatous papules and plagues on the chest	Failed: prednisone, HCQ, MMF, tacrolimus/triamcinolone 0.1% ointment, MTX, IVIG Responded to deucravacitinib 6 mg daily	4 mo into deucravacitinib, patient had marked improvement in heliotrope rash, periorbital edema, and poikilodermatous papules on chest

## Case report

A 65-year-old female with an 8-year history of classic myopathic dermatomyositis presented to clinic for ongoing management. DM workup began in 2017 when she came into clinic with an erythematous, pruritic, maculopapular rash on the upper back and neck for 3 months. Rash did not completely clear with topical hydrocortisone and Medrol dose pack. Punch biopsies were sent for H&E and DIF. Differentials at this time included contact dermatitis, cutaneous lupus erythematosus, photosensitive dermatoses, and dermatomyositis.

**Fig 1:**
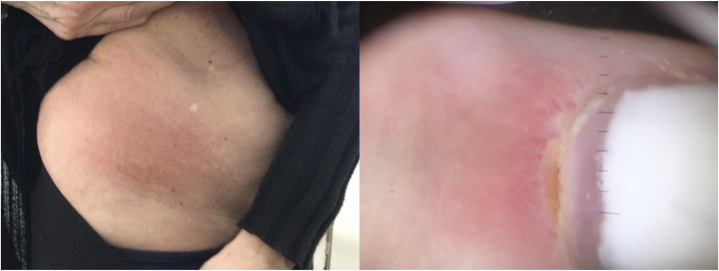
Holster sign (*left*) and nail capillary loop dilation and capillary dropout (*right*), consistent with clinical findings of dermatomyositis.

DM with features of Sjogren syndrome and MCTD was ultimately diagnosed based on: cutaneous manifestations, which included periorbital erythema, shawl sign, Gottron papules, nail capillary loop dilation (Fig 1), serologic findings of ANA (1:160, speckled pattern), myositis panel positive for MI-2 beta and TIF-1γ antibodies, skin biopsy compatible with DM (showing subtle and multifocal interface lymphocytic vacuolar/lichenoid lymphocytic infiltrate with increases of papillary dermal mucin via colloidal iron stain) and DIF consistent but not specific for DM (granular C5b-9 and weaker IgM and C3 deposition and cytoid bodies along BMZ),^6^ alongside complaints of proximal extremity muscle weakness (worse in the shoulder girdle than the hips) and dryness of the mouth and eyes which the patient has kept under control with the use of artificial tears and xylitol lozenges. Other differentials were excluded based on clinical assessment, serologic testing (rheumatologic panel, autoimmune connective tissues panels), and imaging (CT chest, abdomen, pelvis, MRI C-spine).

**Fig 2:**
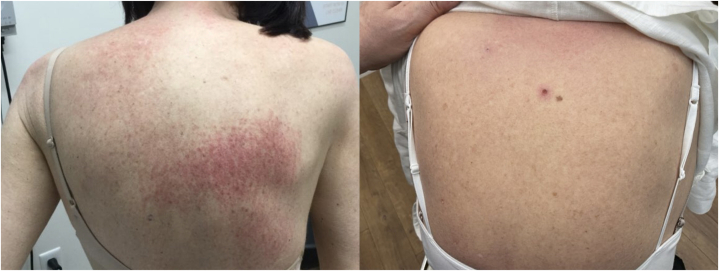
Erythematous rash on upper back (*left*) that improved 11 months into deucravacitinib 6 mg daily (*right*).

Between 2017 and 2024, patient’s cutaneous lesions waxed and waned despite treatments of methotrexate, hydroxychloroquine (HCQ), prednisone, mycophenolate mofetil, and azathioprine. Due to her lack of response to the prior therapies, we theorized the use of deucravacitinib as a treatment, as studies have shown that TYK2-deficient mice demonstrate reduced responses in IL-12/Th1 and IL-23/Th2, inflammatory cytokines and T-helper cells central to DM.^7^ In May 2024, patient was started on off-label deucravacitinib at 6 mg orally every day, alongside her prednisone dose of 10 mg daily per her rheumatologist. At follow up (12/2024), patient’s back was almost completely clear (Fig 2). Near resolution of her rash was maintained, including on recent follow up (4/2025). Patient has tolerated medication well, with no reported side effects or lab abnormalities. She continues to follow up with rheumatology for proximal extremity muscle weakness with normal ADLs (brushing hair, walking upstairs) and will continue to follow up with dermatology for monitoring of long-term efficacy and safety of this treatment. With the current studies of next generation TYK2 inhibitors for DM, there is hope that this regimen will 1 day not be considered off-label, thus making coverage easier.

## Discussion

Augmented interferon (IFN) signaling is a molecular feature of DM, observed in skin, muscle, and peripheral blood.^8^ IFN upregulation induces injury to capillaries, myofibers, and keratinocytes, contributing to disease pathogenesis.^8^^,^^9^ Cutaneous manifestations are particularly associated with elevated type I IFN signaling and type I IFN scores, making type 1 IFN a compelling therapeutic target.^8^

Deucravacitinib is an oral TYK2 inhibitor that is FDA-approved for moderate-to-severe plaque psoriasis. It achieves high selectivity by allosterically binding to the regulatory pseudokinase (JH2) domain of TYK2. This action impedes downstream signaling and attenuates several cytokines implicated in the inflammatory component of DM, including IL-23, IL-12, and type 1 IFNs.^10^ Deucravacitinib is currently undergoing 2 phase 3 clinical trials for systemic lupus erythematosus, POETYK SLE-1 and SLE-2 (NCT05617677 and NCT05620407). Its mechanism suggests it may benefit patients with DM.

The therapeutic relevance of TYK2 inhibition in DM is further substantiated by data from other agents in this class, such as brepocitinib. Brepocitinib is a dual TYK2 and JAK1 inhibitor that has demonstrated promise in the management of DM. Brepocitinib therapy reduces signaling of multiple pro-inflammatory cytokines, including type I and II IFNs, IL-6, and IL-12/IL-23. Its efficacy and safety are under investigation in the ongoing Phase 3 VALOR study (NCT0543726), a 52-week, placebo-controlled trial in patients with dermatomyositis.^11^ Although the results have not yet been peer-reviewed, Roivant released key findings on September 17, 2025. Patients receiving brepocitinib 30 mg achieved a mean Total Improvement Score (TIS) of 46.5, compared with 31.2 in the placebo group (*P* = .0006). Over two-thirds of participants reached at least a moderate response (TIS ≥40), and nearly half achieved a major response (TIS ≥60).^12^ These findings support the role of TYK2 inhibition as a steroid-sparing mechanism for patients with DM.

The patient in this case exhibited marked improvement in the cutaneous involvement of her DM with deucravacitinib therapy, while myopathy remained stable but persisted. These findings, alongside the other 2 cases in Table I, elucidate the mechanistic rationale for TYK2 inhibitors in dermatomyositis and support the potential of deucravacitinib for managing cutaneous disease. Further studies are warranted to contextualize these observations beyond isolated case reports.

## Conflicts of interest

None disclosed.
